# Characterization of early immune responses elicited by live and inactivated vaccines against Johne's disease in goats

**DOI:** 10.3389/fvets.2022.1046704

**Published:** 2023-01-09

**Authors:** Mostafa Hanafy, Chungyi Hansen, Yashdeep Phanse, Chia-wei Wu, Kathryn Nelson, Sophie A. Aschenbroich, Adel M. Talaat

**Affiliations:** ^1^Department of Pathobiological Sciences, School of Veterinary Medicine, University of Wisconsin, Madison, WI, United States; ^2^Department of Microbiology and Immunology, Faculty of Veterinary Medicine, Cairo University, Giza, Egypt; ^3^Pan Genome Systems, Madison, WI, United States; ^4^Research Animal Resources Center, University of Wisconsin-Madison, Madison, WI, United States

**Keywords:** *paratuberculosis* (MAP), Johne's disease, vaccine, immunity, live vaccine, inactivated vaccine

## Abstract

Mycobacterium avium subspecies paratuberculosis (*M. paratuberculosis*) is the causative agent of Johne's disease, a chronic debilitating condition affecting ruminants causing significant economic losses to the dairy industry. Available inactivated vaccines are not effective in controlling the disease and vaccinated animals can continue to infect newly born calves. Recently, we have shown that a live-attenuated vaccine candidate (pgsN) is protective in goats and calves following challenge with virulent strains of *M. paratuberculosis*. To decipher the dynamics of the immune responses elicited by both live-attenuated and inactivated vaccines, we analyzed key immunological parameters of goats immunized through different routes when a marker-less pgsN vaccine was used. Within a few weeks, the inactivated vaccine triggered the formation of granulomas both at the site of inoculation and in regional lymph nodes, that increased in size over time and persisted until the end of the experiment. In contrast, granulomas induced by the pgsN vaccine were small and subsided during the study. Interestingly, in this vaccine group, histology demonstrated an initial abundance of intra-histiocytic mycobacterial bacilli at the site of inoculation, with recruitment of very minimal T lymphocytes to poorly organized granulomas. Over time, granulomas became more organized, with recruitment of greater numbers of T and B lymphocytes, which coincided with a lack of mycobacteria. For the inactivated vaccine group, mycobacterial bacilli were identified extracellularly within the center of caseating granulomas, with relatively equal proportions of B- and T-lymphocytes maintained across both early and late times. Despite the differences in granuloma-specific lymphocyte recruitment, markers for cell-mediated immunity (e.g., IFN-γ release) were robust in both injected pgsN and inactivated vaccine groups. In contrast, the intranasal live-attenuated vaccine did not elicit any reaction at site of inoculation, nor cell-mediated immune responses. Finally, 80% of animals in the inactivated vaccine group significantly reacted to purified protein derivatives from *M. bovis*, while reactivity was detected in only 20% of animals receiving pgsN vaccine, suggesting a higher level of cross reactivity for bovine tuberculosis when inactivated vaccine is used. Overall, these results depict the cellular recruitment strategies driving immune responses elicited by both live-attenuated and inactivated vaccines that target Johne's disease.

## Introduction

Johne's Disease (JD) is caused by infection with *Mycobacterium avium subspecies paratuberculosis* (*M. paratuberculosis*) in ruminants characterized by chronic diarrhea and weight loss (1). The disease is also accompanied with significant decreases in milk yield and up to 20% mortality in infected herds resulting in large economic losses estimated around $400 million/year to the dairy industry in the US alone (2). Calves are usually infected during the first weeks of life, mostly from infected adult cows in the herd that shed viable *M. paratuberculosis* in feces. Infections remain subclinical until affected animals become immunosuppressed (e.g., during calving, transportation, etc.), at which point chronic diarrhea becomes prevalent (3). Immunization with inactivated, whole-cell vaccine, like Mycopar^®^ within 35 days of birth, is the only licensed type of vaccines for dairy cows, sheep, and goats (4). While inactivated vaccines decreased the number of *M. paratuberculosis*-positive animals, they do not prevent fecal shedding allowing spread of infection within infected herds (5). The inclusion of paraffin oil in some of the inactivated vaccines (e.g., Mycopar^®^) usually enhance the formation of a large granuloma at the site of injection (6, 7), leading to purulent exudate and fistulation in some animals (8). To improve vaccine safety and protective efficacy, we constructed a vaccine candidate (referred to herein as pgsN) using homologous recombination to delete the *lipN* gene from *M. paratuberculosis* K10 strain (9). When tested in both the goat (8) and cow (10) models of Johne's disease, the pgsN vaccine prevented fecal shedding of *M. paratuberculosis* and induced produced robust T-cell immunity. In this report, we generated a second generation pgsN vaccine by rescuing the antibiotic marker (marker-less pgsN) and evaluated the safety and early immune responses of the version of the pgsN vaccine in goats following subcutaneous (SC) and intranasal (IN) immunizations, in anticipation of developing an easier strategy for vaccine delivery.

In 2000, the National Research Council indicated the dire need to fill several knowledge gaps associated with the pathophysiology, immunology and control of JD (11). Several attempts to develop new JD vaccines have been made, including subunit and live-attenuated vaccines. An Hsp70 subunit vaccine administrated subcutaneously elicited both antigen-specific antibody responses (12) and mononuclear cell proliferations (13), but did not stimulate protective Th1-type immune responses in cattle (12). A cocktail of four polypeptides [Ag85A, Ag85B, superoxide dismutase (SOD) and Map74F] encoded by *M. paratuberculosis*, induced significant antigen-specific lymphoproliferations and IFN-γ secretion in vaccinated goats, suggesting the induction of Th1-immune responses (14). With the high expenses of producing a protein cocktail for a subunit vaccine to be used in animals (15), our group and others explored alternatives including live-attenuated vaccines. An auxotroph of *M. paratuberculosis* with deleted *leuD* gene was found to elicit protective immunity against JD in goats (16). So far, the *leuD* mutant was not evaluated in cattle, the main target host for Johne's disease. To find mutants that could serve as vaccines, we and others screened *M. paratuberculosis* transposon mutant libraries that identified a few candidates (17–21). Using targeted deletions in a virulent strain of *M. paratuberculosis*, mutants of both *relA* and *pknG* genes were evaluated in calves and *relA* provided protective immunity in a goat challenge model (22). Additional potential vaccine candidates were identified using transcriptional profiling, which targeted several virulence factors encoded in *M. paratuberculosis* including *lipN* (encoding a possible lipase/esterase), *sigH* (an alternative sigma factor important for the stress response) and *sigL* (an alternative sigma factor involved in virulence) (9, 17, 23). Subsequent analyses demonstrated that *M. paratuberculosis* isogenic mutants of *lipN* (pgsN), *sigL* and *sigH* provided protective immunity by reducing tissue lesions and tissue colonization against challenge with *M. paratuberculosis* in several animal models of paratuberculosis including mice, goats and cows (8, 10, 24, 25). However, how these candidates modulate host immunity to develop protection against *M. paratuberculosis* remains a significant knowledge gap that need to be addressed.

In this report, we generated a marker-less construct of *M. paratuberculosis* Δ*lipN* mutant (pgsN) (9) where the *hyg* gene was rescued and the removal of the marker gene was confirmed by whole genome sequence analysis. The safety and immunogenicity profiles of the new construct were evaluated in goats to further characterize early immune responses generated by this vaccine following both SC and IN immunization in comparison to the SC immunization with inactivated Mycopar vaccine. Comparative analysis of the new pgsN construct showed a similar immune profile to the parent strain with the demonstrated safety and tolerance by goats throughout the experiment. More importantly, the psgN vaccine was associated with an induction of T lymphocytes that were few in numbers in the early time post vaccination and increased by the end of the experiment at 6 months post vaccination (MPV), unlike immunization with the inactivated vaccine. On the contrary, large, caseating and mineralized granulomas were developed early on following injection with the inactivated vaccine that continued until the end of the experiment where cross reactivity to bovine tuberculosis test was demonstrated only in this group.

## Materials and methods

### Generation of a marker-less vaccine

The vaccine candidate pgsN (*M. paratuberculosis* Δ*lipN*) used in this study was generated as detailed before (9) with some modification. In the current study, the hygromycin resistance gene cassette was removed using γδ-resolvase as detailed before (26), to improve the utility for field application. The vaccinal strain was grown in modified Middlebrook 7H9 (BD Biosciences, Sparks, MD, USA) (27) to avoid growing in media with animal sources, another requirement for field application of animal vaccines. To ensure the removal of the hygromycin cassette, whole genome sequencing was performed on pgsN genomic DNA using Illumina HiSeq2500 and PacBio SMRT (28). The SPAdes 3. 15.1 software was used for genome assembly. Genome scaffolding was done using Medusa v1.3 (29). The single contig yielded was aligned to the *M. paratuberculosis* strain K10 (Accession number AE016958.1) using Bacteria and Viral bioinformatics resource center (https://www.bv-brc.org). The comprehensive genome analysis tool was used to annotate and compare the finished genome to *M. paratuberculosis* K10. The new construct was then propagated in modified Middlebrook 7H9 (27) until it reached OD_600_ = 1.0. Then, the culture was centrifuged at 3,200 x g/30 min. Bacterial pellet was resuspended 1/10^th^ of its original volume in 0.9% NaCl and 10% glycerol solution and stored at−80 degrees until used for vaccination.

### Study design

**Figure 1:**
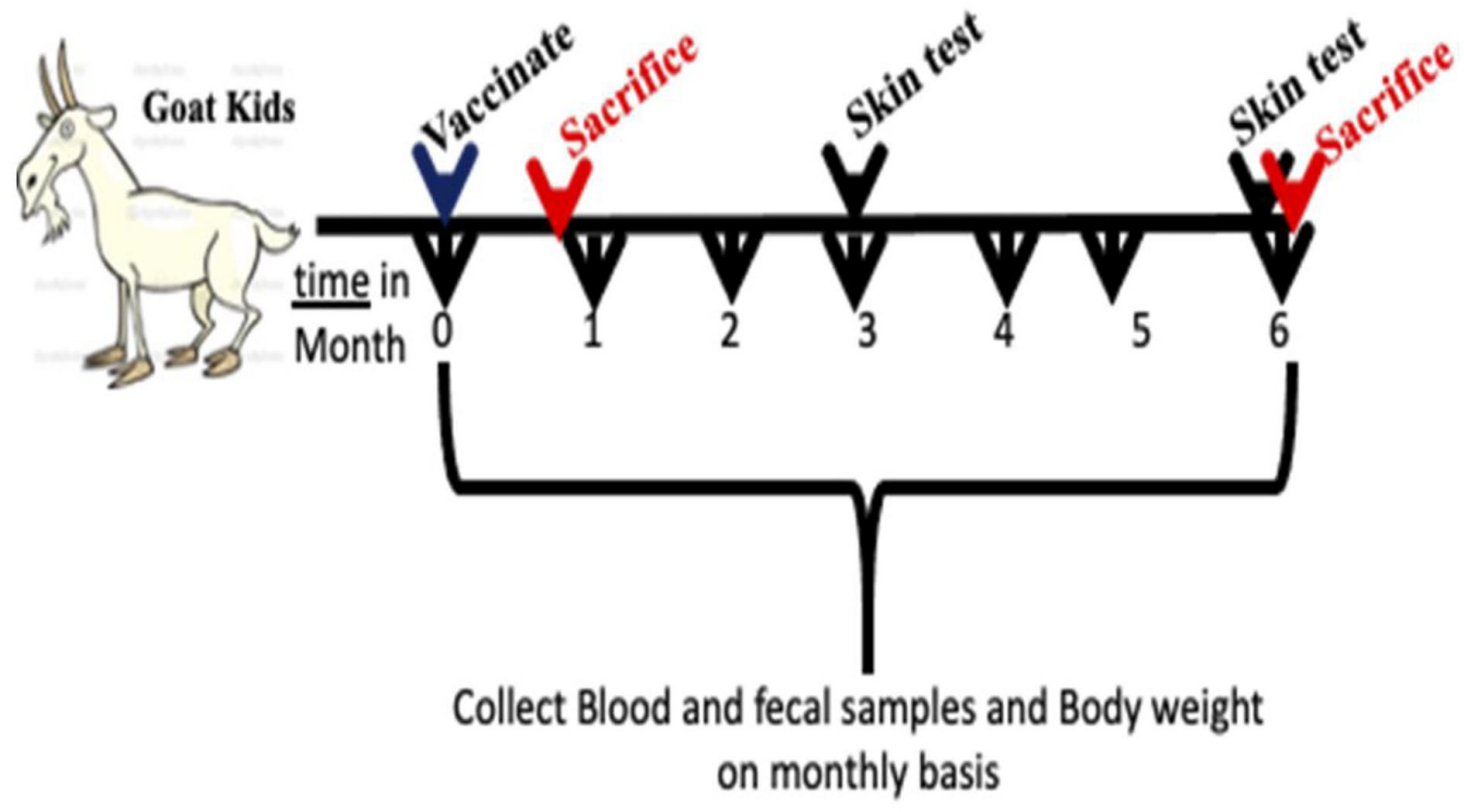
Sketch for experimental design used in this study. Goat kids at 28 days of age were vaccinated by subcutaneous injection or intranasal and monitored for 6 months post vaccination. Two sacrifices were conducted at 3 weeks then at 6 months post vaccination. Feces, blood and saliva were collected once a month throughout the experiment timeline.

A total of 32 male goat kids were purchased from a local farm in Wisconsin, USA. Serum samples from the pregnant dams were tested for paratuberculosis by ELISA (Paracheck II; Biocor Animal Health, Omaha, NE, USA) and kids selected for the study were from JD negative dams that gave birth within 7 days of each other for minimal age variance in groups. In addition, environmental samples (feces and drinking water from animal pens) were collected and cultured for isolation to evaluate the presence of mycobacteria in the herd, as a whole (Data not shown). All animal care and experimental procedures were conducted in compliance with the protocols approved by the Institutional Animal Care and Use Committee (IACUC), University of Wisconsin-Madison. Goat kids (*N* = 8/group) were assigned to one of four groups, with each group receiving either phosphate buffered saline (PBS) injected subcutaneously (PBS, SC), pgsN vaccine delivered subcutaneously (pgsN-SC), pgsN vaccine delivered intranasally (pgsN-IN) and Mycopar^®^ inactivated vaccine administered subcutaneously (inactivated vaccine). The commercial Mycopar^®^ (Boehringer Ingelheim Vetmedica, Inc., St. Joseph, MO, USA) was inoculated subcutaneously as directed by the manufacturer. Blood, fecal and saliva samples were collected on monthly basis (Figure 1). Individual rectal body temperature was measured using a digital thermometer. Following subcutaneous injection of vaccines, skin thickness at the site of injection was assessed using a digital measuring device. All measurements for vaccine safety (temperature, skin thickness, body weight) were collected 1 day prior to vaccination and continued up till 3 days post vaccination (1, 2, and 3 dpv) and then once per month for the remaining 6 months. Animals were regularly inspected by animal care staff and board-certified veterinarians at the University of Wisconsin-Madison.

### Vaccine preparation

Aliquots of frozen marker-less pgsN vaccine were thawed, pelleted and then suspended in PBS and counted on Middlebrook 7H11 (BD Biosciences, Sparks, MD, USA) agar plates supplemented with 2 μg/ml mycobactin J (Allied Monitor, Fayette, MO, USA), and 10% ADC (2% glucose, 5% bovine serum albumin fraction V, and 0.85% NaCl) before every vaccine administration. Vaccine doses were adjusted in PBS to obtain a dose of 10^9^ CFU/animal, then passed through a 27G needle attached to 10 ml syringe to disaggregate any clumps. The Quil-A adjuvant (Desert King, San Diego, CA, USA) was added to the vaccines doses at the rate of 100 μg of QuilA/ml of vaccine. One ml of the pgsN vaccine preparation was injected in the right shoulder of each kid. Similarly, kids in the Mycopar^®^ group were injected with the vaccine per manufacturer instruction. For intranasal (IN) immunization, the 1 ml pgsN (without adjuvant) was delivered using syringe atomization device (Teleflex, Morrisville, NC, USA). All kids were vaccinated at 28 days of age.

A subset of animals (*N* = 3) representing all experimental groups (pgsN-SC, pgsN-IN, Inactivated-SC, PBS) were euthanized at 3 weeks post vaccination (WPV) while the rest were sacrificed at 6 months post vaccination (MPV). Samples collected at the early time point (3 WPV) were used to examine vaccine safety and induction of early immune responses in vaccinated animals. At necropsy, the overall health condition of goats was evaluated and any apparent gross lesions or evident granuloma formation were recorded for each animal. Tissue samples were collected on ice from or the following: liver, spleen, lung, three different sections of duodenum, three different sections of jejunum, the entire ileum, ileocecal valve, ipsilateral prescapular lymph nodes, ileocecal lymph nodes, hepatic lymph nodes, and skin from the site of injection. A section of each tissue listed above that also were fixed in 10% neutral buffered formalin (NBF) for subsequent histopathologic evaluation.

###  Specimen culture

To determine mycobacterial count in tissues, 2–3 g of internal organs (liver, spleen, lung, ipsilateral prescapular lymph nodes, ileocecal and hepatic lymph nodes) were individually homogenized after adding 10 ml of sterile PBS to each sample in a Whirl-Pack bag for 10 min, on high, using a stomacher (Seward USA, Davie, FL, USA). Tissue homogenates were centrifuged at 1000 x g for 15 min, then the pellet was resuspended in 1 ml of sterile PBS. To determine mycobacterial count in intestinal sections, 2–3 g of each organ were homogenized separately in 10 ml of 0.75 hexadecyl pyridinium chloride (HPC) (Sigma-Aldrich, St. Louis, MO, USA) for 10 min and incubated for 4 h at room temperature before HPC was pipetted out and an equal amount of sterile water was added. Tissue homogenates were centrifuged at 1000 x g for 15 min, and the pellet was suspended in 1 ml of Middlebrook 7H9 broth supplemented with 2 μg/ml mycobactin J, 10% ADC (2% glucose, 5% bovine serum albumin fraction V, and 0.85% NaCl), vancomycin (1 mg/ml), amphotericin B (0.5 mg/ml) and Nalidixic acid (1 mg/ml) (VAN) and incubated at 37°C for 24 h in shaking incubator before plating.

For fecal cultures, 2–3 g of feces were homogenized in 30 ml of sterile water and allowed to passively sediment for 20 min, with subsequent collection of sediment-free supernatants. Equal amount of 1.5% HPC were added to the sediment-free supernatant and incubated for 4 h at room temperature. HPC- treated samples were centrifuged at 1000 x g for 15 min and pellets were resuspended in Middlebrook 7H9 supplemented with mycobactin J and antibiotics (see above) for 24 h before plating on Middlebrook 7H11 agar supplemented with the same concentrations of mycobactin J and antibiotics. For saliva, 1 ml of saliva was scaled up to 5 mL using sterile distilled water before adding an equal volume of 1.5% of HPC and incubating for 4 h at room temperature. Samples were centrifuged at 1000 x g for 15 min and resuspended in Middlebrook 7H9 + Mj + VAN, as before. For all plating, aliquots from final suspensions and diluted samples were plated in duplicates on Middlebrook 7H11 supplemented with 2 μg/ml mycobactin J, 10% ADC and VAN. All plates were incubated at 37°C for 8–12 weeks before counting colonies.

### Histology and immunohistochemistry

For histology, tissue samples from harvested organs were routinely processed, paraffin embedded, sectioned, and stained with hematoxylin and eosin (H&E) for evaluation. Tissue sections stained with H&E or Fite's acid fast stain were evaluated for the presence of granuloma formation and to confirm intralesional mycobacteria, respectively. Immunohistochemistry was performed by the University of Wisconsin-Madison, School of Veterinary Medicine, histology laboratory, on tissues with histologically evident, lymphocyte-poor and lymphocyte-rich granulomas and was utilized to further confirm differential lymphocyte recruitment identified by H&E stain. For these immunohistochemical analyses, CD3 (DAKO, M725401-2, dilution 1:200, unconjugated, monoclonal) and CD20 (Fisher, RB9013P, dilution 1:400, unconjugated, polyclonal) antibodies were utilized to further highlight T and B lymphocytes, respectively, and analyses were developed using the Po-Link 2 Plus HRP Broad for DAB Bulk Kit Polymer detection kit (GBI Labs, D41-110). A subjective scoring system was established to characterize the extent of lymphocyte recruitment to granulomas and included a score 1 for minimal numbers, 2 for small numbers, 3 for moderate numbers, and 4 for high numbers of lymphocytes; the same scoring system was applied to both routinely stained sections and to those subjected to immunohistochemistry. All histologic and immunohistochemical studies were conducted by a board-certified veterinary anatomic pathologist blinded to experimental conditions.

### Measurement of cell-mediated immunity

The single comparative intradermal skin test (SCIST) was performed by a board-certified veterinarian to all goats at 3 MPV and at 6 MPV. Standard bovine purified protein derivatives (PPD-b), and Johnin (PPD-j) were purchased from the National Veterinary Services Laboratory (Ames, IA, USA). A volume of 0.1 ml of each PPD was administered by intradermal injection in the cervical region using standard tuberculin syringes. Injection sites were pre-measured and marked with a colored marker for easier determination of the injection site location. The response to the PPD injections (skin thickness/induration) was measured using digital calipers at 72 h post-injection.

For the IFN-γ assay, 5 mL of blood samples were collected from the jugular vein into vacutainer tubes containing sodium EDTA anticoagulant to isolate peripheral blood mononuclear cells (PBMCs). Each blood sample was diluted in equal amount of RPMI supplemented with 1% fetal bovine serum (FBS), penicillin (100 IU/mL) and streptomycin (100 μg/mL) (this media will subsequently be referred to as “R1”). The diluted blood was layered over 7.5 ml of Histopaque-1077 (Sigma-Aldrich, St. Louis, MO, USA) and centrifuged at 400 x g for 30 min. Cell layers were pipetted out to add an equal volume of R1 and centrifuged at 400 x g for 10 min, supernatant was removed, and 1 ml of 1 x BD Parma Lyse™ RBC lysis buffer (BD Biosciences, San Jose, CA, USA) was added and incubated for 1 min at room temperature before adding 5 mL of R1 and centrifugation as before. The cells were suspended in 2 mL of RPMI supplemented with 10% FBS, penicillin (100 IU/mL) and streptomycin (100 μg/mL) (this medium will subsequently be referred to as “R10”) and counted using a hemocytometer. Extracted PBMCs from each animal were diluted to a concentration of 10^7^ cells/ml in R10 and 100 ul of each sample was dispensed into a 96 well round bottom plates. Each sample was represented by 4 wells where 2 wells were stimulated with PPD-j 10 ug/ml + IL-2 (BD Biosciences, Franklin Lakes, NJ) (400 u/ml) and the other 2 wells were stimulated with IL-2 only (negative control). Cells were incubated for 72 h before harvesting the supernatants for the IFN- γ ELISA assay (BOVIGAM, Prionics, Omaha, NE, USA) as described before (8). The results of IFN- γ was expressed as ELISA index calculated as before (9).

### Humoral immune response

A total of 5 mL of whole blood samples were collected from the jugular vein on monthly basis from each animal using vacutainer tubes (Becton Dickinson and Co., Franklin Lakes, NJ). Serum samples were separated by centrifugation and anti-*M. paratuberculosis* antibodies were estimated using Paracheck 2 ELISA kit (Prionics, Omaha, NE, USA) according to the manufacturer's instructions.

### Statistical analysis

Statistical significance of the differences between groups in each assay was measured using either one-way analysis of variances (ANOVA) with multiple comparisons using Fisher's LSD or student's *t*-test wherever the test is appropriate, a *p*-value ≤ 0.05 was considered significant. All statistical analysis was conducted using GraphPad Prism 7 (GraphPad Software, Inc., La Jolla, CA, USA).

## Results

### Generation of marker-less pgsN vaccine

**Figure 2:**
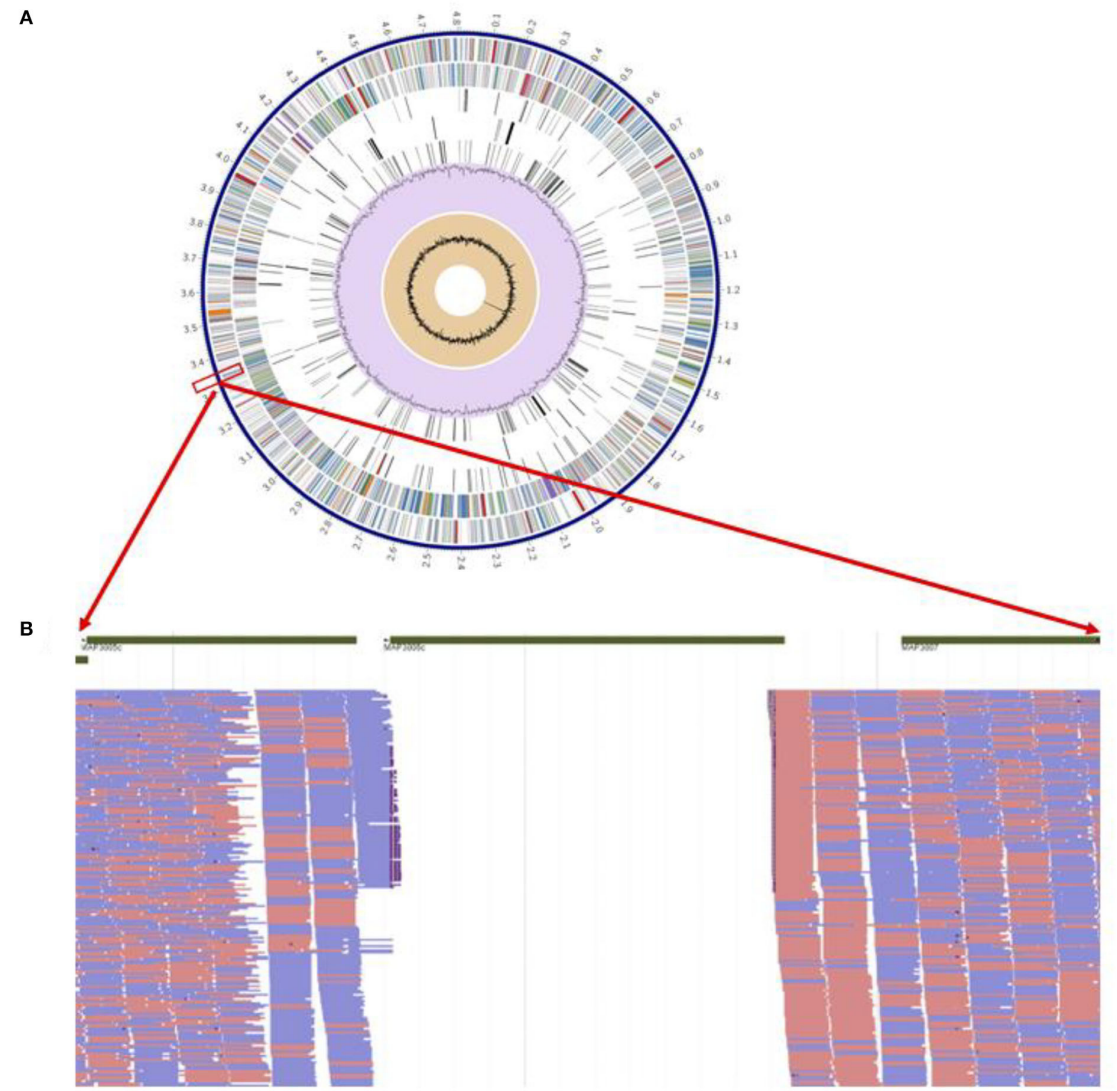
Genome-wide analysis of live attenuated vaccine pgsN. **(A)** Annotated genes on the aligned *M. paratuberculosis* Δ*lipN* showing from outer to inner; scaffold of *M. paratuberculosis* k10, coding DNA sequences (CDS) on forward, CDS on reverse, non-CDS features, non-synonym mutations on forward CDS, non-synonym mutations on reverse CDS, GC content and GC skew. **(B)** Magnification of short reads aligned to areas flanking the *lipN* gene to confirm successful knockout of *lipN* by the absence of aligned reads.

The pgsN mutant was generated by homologous recombination in the *M. paratuberculosis* K10 wild type strain, as described before (9). However, we sought to rescue the hygromycin cassette (*hyg*^*r*^) (26) to avoid spreading antibiotic resistance genes in vaccinated animals should the vaccine be administered in the field. Following removal of the *hyg*^*r*^ gene, Illumina whole genome sequence analysis confirmed the *lipN* deletion and the removal of the *hyg*^*r*^ cassette (Figure 2). As expected, assembly of sequenced reads were able to identify a whole genome size of ~4.8 MB represented by a single contig with GC content that reached 69.3%, similar to the isogenic *M. paratuberculosis* K10 wild type strain as shown in Table 1 (30). BLAST sequence analysis of the assembled genome compared to the genome of *M. paratuberculosis* K10 confirmed the deletion of *lipN* gene and the removal of *hyg*^*r*^ cassette.

**Table 1:** Assembly details for *M.ap* Δ*LipN* genome.

Contigs	1
GC content	69.31
Plasmids	0
Contig L50	1
Genome length	4,807,932 bp
Contig N50	4,807,932

### The live attenuated pgsN vaccine is safe

**Figure 3:**
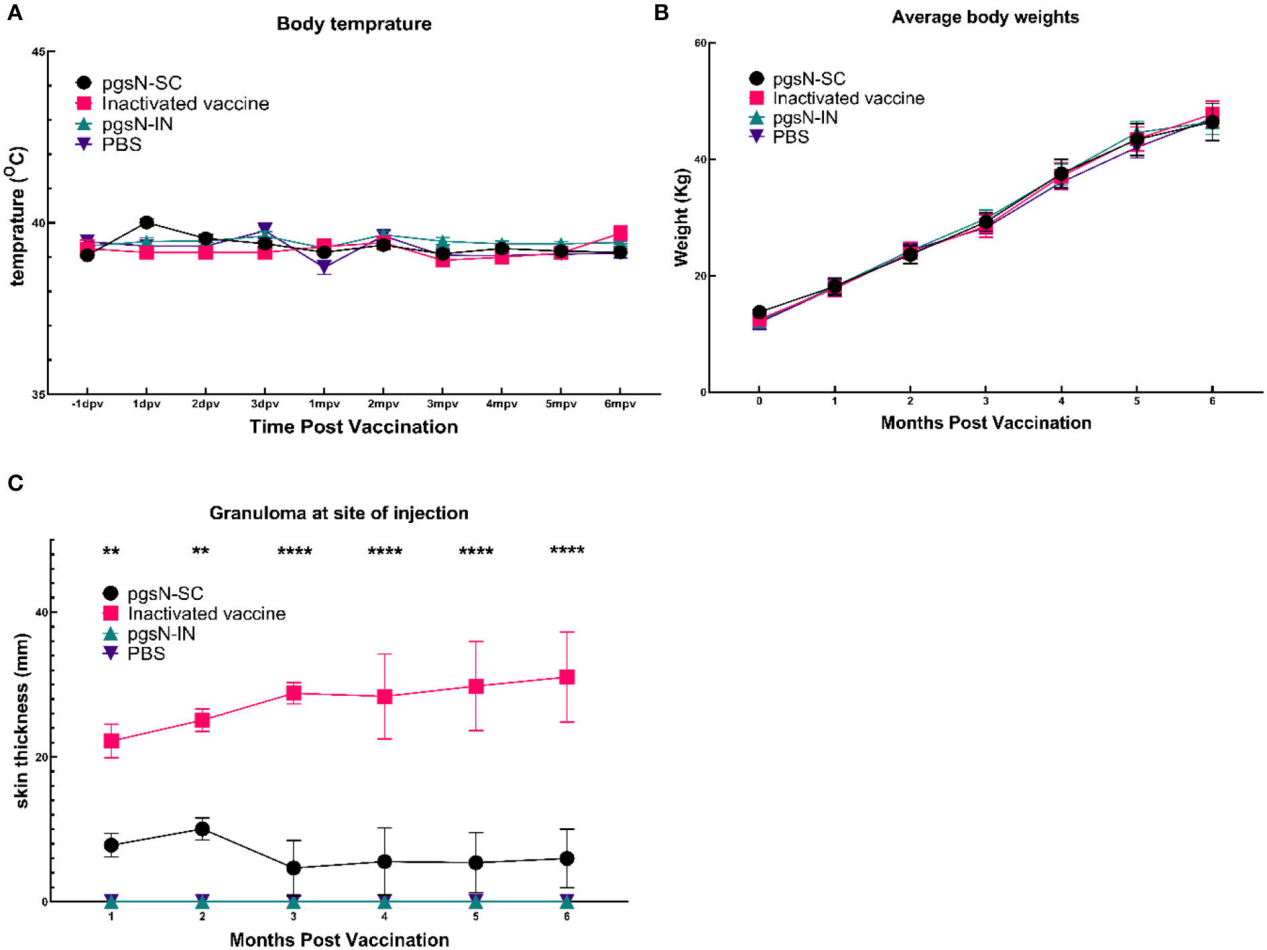
Safety of pgsN vaccine. **(A)** The average body temperatures at 1 day before vaccination, the first 3 days post vaccination as well as every month post vaccination. **(B)** The average weights for each group throughout the study timeline. **(C)** The average size of the granuloma at the site of injection. Bars represent the standard error asterisks indicate statistical significance between pgsN-SC and inactivated vaccines using Student *t*-test. **, *p*-value < 0.01; ***, *p*-value < 0.001; ****, *p*-value < 0.0001.

The pgsN vaccine was evaluated in goats following immunization by either SC or IN routes up to 6 MPV. All animals were monitored for key animal health parameters such as body weight, temperature fluctuations, or development of any signs of illness. All experimental animals survived throughout the study period, and body temperatures were within a normal range at all sampling timepoints (Figures 3A, B). We also monitored the site of vaccination to assess potential granuloma development or any other vaccine-induced skin lesions. As expected, the injection site increased in size for both live-attenuated pgsN-SC and inactivated-SC vaccine groups administered *via* the SC route. For the pgsN-SC group, the maximum lesion size reached ~ 8 cm at 2 MPV (Figure 3C), which started to decline by 3 MPV to reach ~2 cm by the experimental endpoint. For the inactivated-SC vaccine group, granuloma size steadily increased during the study for all animals, from ~20 cm at 1 MPV, to ~30 cm by 6 MPV. In addition, granulomas from two animals in the inactivated-SC vaccine group ruptured and required surgical debridement at 4 MPV. The progressive nature of the granulomas and subsequent rupture highlighted some of the drawbacks associated with the inactivated vaccine, which was not seen in the live-attenuated vaccine groups.

A key aspect of vaccine safety is persistence of the vaccine strain. To confirm pgsN safety, we monitored shedding of the vaccine strain in saliva and feces in addition to collecting tissues. Culturing of saliva and fecal samples from pgsN-vaccinated animals did not yield any mycobacterial colonies, suggesting it is unlikely that shedding of pgsN occurs for either route. At 3 WPV, three animals were sacrificed from each of the four study groups to monitor live vaccine persistence in organs. Although all tissues were cultured for bacterial isolation, only the site of injection and prescapular lymph nodes of the pgsN group inoculated *via* the SC route yielded acid -fast bacilli (AFB) in the range of 10^2^-10^5^ cfu/g of tissues at 3 WPV. At 6 MPV, culturing of harvested tissues from the remaining animals in all live-attenuated vaccine groups revealed no mycobacterial colonies, indicating that pgsN is cleared from animals by 6 MPV, similar to culturing of tissues from animals in the inactivated vaccine group that yielded no colonies, as expected. Overall, the live-attenuated vaccine given *via* SC or IN routes had little or no negative effect on animal health, while the inactivated-SC vaccine induced large granuloma formation that required surgical intervention in some animals.

### Intralesional immunologic responses to immunization by live and inactivated vaccines

To understand the nature of early immune responses generated by the live-attenuated pgsN or inactivated vaccines, we sacrificed animals and harvested organs at 3 WPV and 6 MPV. Histologic sections of harvested organs were examined by routine and histochemical staining for the presence of granuloma formation and mycobacterial bacilli. As expected, sections taken from internal organs of animals in the PBS control group at 3 WPV or 6 MPV did not reveal any histologic lesions. Granuloma formation was likewise not observed in the pgsN-IN group. In the pgsN-SC or inactivated-SC vaccine groups, visceral organs such as liver, spleen, intestine, and lung had no significant histologic changes or intralesional bacilli at all examined times. For animals in the pgsN-SC or inactivated-SC vaccine groups with grossly evident subcutaneous granulomas, tissue sections from these injection sites were additionally subjected to immunohistochemistry for CD3 and CD20, which are markers for T and B lymphocyte populations, respectively, to further analyze and confirm the cellular composition of post-vaccinal granulomas.

**Figure 4:**
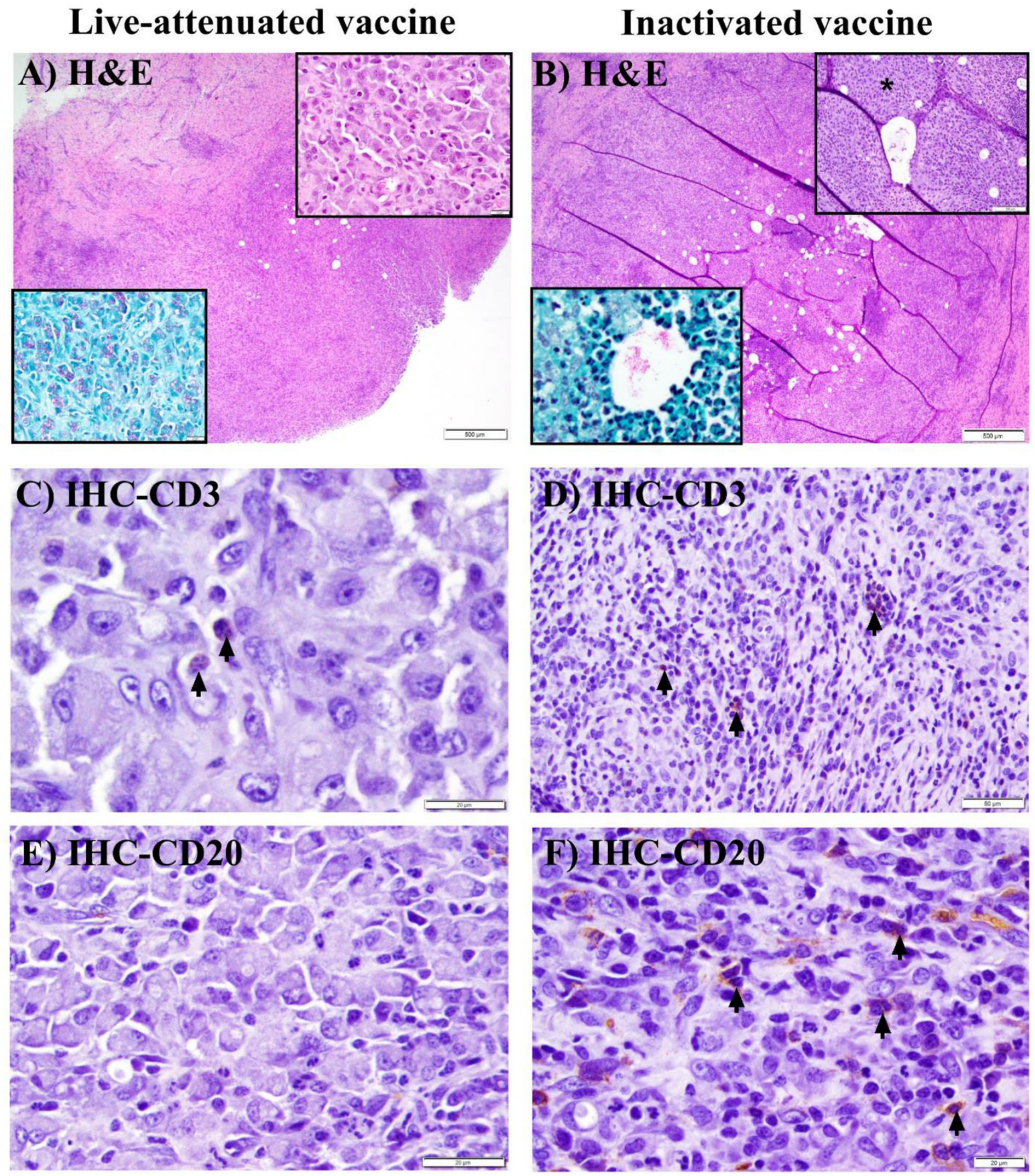
Histopathology of granuloma formation in goats after immunization with live-attenuated or inactivated vaccines at 3 weeks post vaccination. **(A, B)** Hematoxylin and eosin (H&E) stained sections from subcutaneous granulomas at the site of injection of SC-vaccinated goats demonstrating granulomatous inflammation **(A)** to granuloma formation **(B)** (bars = 500 μm). **(A)** Top (H&E) inset shows sheets of epithelioid macrophages, while the bottom (acid-fast stain) inset shows myriad intra-histiocytic acid-fast bacilli (AFB). **(B)** Top (H&E) inset displays degenerate neutrophils **(asterisk)** at the center of the granuloma, with intralesional bacilli in both H&E and acid-fast stain insets. **(C, D)** Immunohistochemical (IHC) staining of the sections in **(A, B)** with arrows pointing to minimal **(C)** or small **(D)** numbers of CD3 + T lymphocytes (bars are 20 & 50 μm, respectively). **(E, F)** IHC staining of the sections in **(A, B)** with arrows pointing to small to moderate numbers of CD20+ B lymphocytes in F, with no detectable B lymphocytes in E (bars = 20 μm).

At 3 WPV, all animals receiving the pgsN and inactivated vaccines developed injection-site specific granulomas with intralesional AFB (Figures 4A, B acid-fast stain insets). In the inactivated-SC vaccine group, granulomas were additionally observed in regional (prescapular) lymph nodes in 1/3 animals at this time point. In general, pgsN-associated vaccination site specific granulomas were often poorly organized (Figure 4A), with formation of dense sheets of epithelioid macrophages (Figure 4A, top inset), and virtually all macrophages had abundant cytoplasmic bacilli, as shown by acid-fast staining (Figure 4A, acid-fast stain inset). In contrast, in animals receiving the inactivated-SC vaccine, granulomas at injection sites were more organized (Figure 4B), with small, neutrophilic (Figure 4B H&E inset, asterisk) to minimally mineralized caseating centers in which extracellular AFB were present (Figure 4B, H&E and acid-fast stain insets). Furthermore, all animals vaccinated with pgsN-SC had very minimal lymphocytes representing CD3+ T cells (Figure 4C), with virtually no CD20+ B cells detected (Figure 4E) within granulomatous areas. In contrast, in the inactivated-SC vaccine group, granulomas were rimmed by relatively higher lymphocyte numbers, with small numbers of CD3 + T (Figure 4D) and small to moderate numbers of CD20 + B cell lymphocytes (Figure 4F).

**Figure 5:**
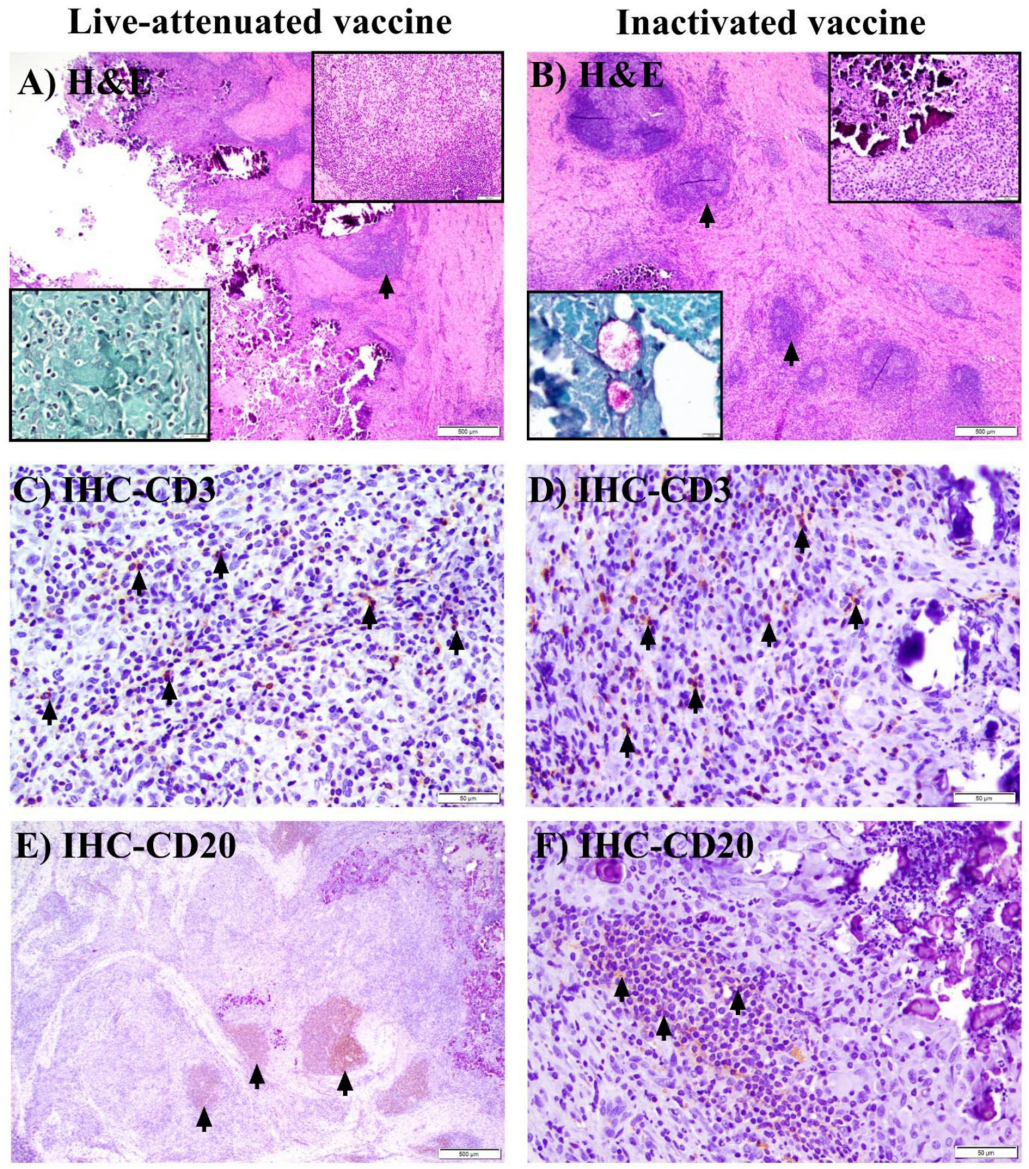
Histopathology of granuloma formation in goats after immunization with live attenuated or inactivated vaccine at 6 months post vaccination. **(A, B)** Hematoxylin and eosin (H&E) stained sections from subcutaneous granulomas at the site of injection of a single SC-vaccinated goat (no other goats in the live-attenuated cohort had grossly or histologically evident granulomas, while all goats in the inactivated vaccine group had subcutaneous granulomas). Arrows point to lymphoid nodular aggregates at the periphery of granulomas, with peri-granuloma lymphocyte recruitment further highlighted by the top (H&E) inset **(A)** (bar = 100 μm) and top (H&E) inset **(B)** (bar =50 μm). The bottom (acid-fast stain) inset in **(A)** shows lack of AFB in subcutaneous granulomatous areas **(A)** in the single pgsN-vaccinated animal; in contrast, extracellular bacilli are present within the caseating center of subcutaneous granulomas in the inactivated vaccine group [**(B)**; acid-fast stain, bottom inset]. **(C, D)** Immunohistochemistry (IHC) staining of the sections in **(A, B)** with arrows highlighting CD3 + T lymphocytes located at the periphery of granulomas (bars = 50 μm). **(E, F)** IHC staining of the sections in **(A, B)** with arrows pointing to CD20 + B lymphocytes forming large nodular aggregates that peripheralize granulomas (bars = 500 and 50 μm, respectively).

At 6 MPV, only 1 animal in the pgsN-SC group had a grossly evident (~2 cm) skin induration, which histologically corresponded to well-organized, heavily centrally mineralized, caseating granulomas with absence of intralesional bacilli by H&E (Figure 5A) or acid-fast stains (Figure 5A, acid-fast stain inset). These granulomas were generally surrounded by a thick rim of moderate numbers of CD3 + T lymphocytes (Figure 5C), with high numbers of CD20 + B lymphocytes often forming large nodular aggregates (Figure 5E). In the inactivated vaccination group, at 6 MPV, in addition to the site of injection, granulomas also developed in additional prescapular lymph nodes to affect 5/5 of the animals, with progressive involvement of mesenteric lymph nodes in 3/5 animals. Animals in this vaccine group had larger granulomas that progressed to developing heavily mineralized, large caseating cores (Figure 5B), as compared to granulomas at 3 WPV with this vaccine. Additionally, among these animals, 2/5 had persistence of AFB within granulomas at injection sites (Figure 5B AFB inset) and prescapular lymph nodes (data not shown). Moreover, the inactivated vaccine group had similar proportions of CD3+T (Figure 5D) and CD20+B (Figure 5F) lymphocytes recruited to the periphery of granulomas at all affected sites (and in similar proportions to the 3 WPV time) with formation of lymphoid nodular aggregates at the periphery of granulomas at the 6 MPV time point (Figure 5B). In general, both T and B lymphocytes were present in the pgsN-SC and inactivated vaccine groups by 6 MPV.

### The pgsN vaccine elicits unique and specific immune responses

**Figure 6:**
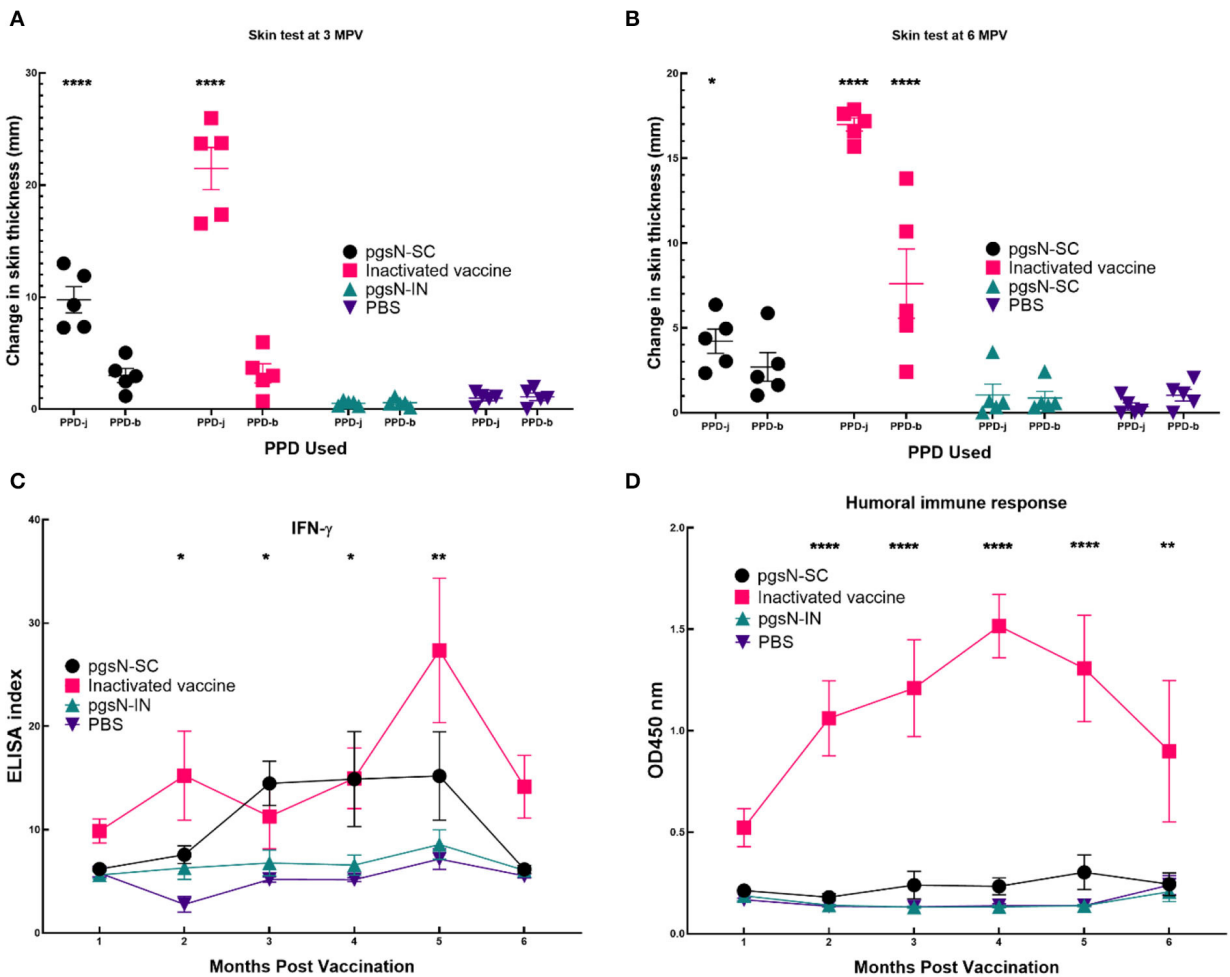
Immune responses to vaccination. Evaluation of cell Mediated Immunity as measured by reaction to intradermal skin test at **(A)** 3 and **(B)** 6 months post vaccination (5 animals/group). Each point in **(A, B)** represents one animal's difference in skin thickness at the PPD site of injection at 0 and 72 h post injection. Purified protein derivative from both *M. paratuberculosis* (PPD-j) and *M. bovis* (PPD-b) were used to inject animals. Bars represent the standard error measurements for each group. **(C)** Measurement of IFN-γ levels in vaccinated goat groups. Peripheral blood mononuclear cells (PBMCs) were isolated from whole blood and stimulated with PPD-j for 72 h. IFN-γ levels in culture supernatants were determined using a Bovigam ELISA kit. Data are expressed as an ELISA index, with error bars represent the standard error. **(D)** Humoral Immune responses as measured by commercial ELISA from sera collected from immunized animals. Asterisks indicate statistical significance between each group immunized with subcutaneous injection compared to PBS using ANOVA. *, *p*-value < 0.05; **, *p*-value < 0.01; ****, *p*-value < 0.0001. In **(D)** only inactivated vaccine gave significant difference.

In a separate analysis, we used SCIST to examine recall immune responses in vaccinated animals. At both 3 and 6 MPV, animals from the pgsN-SC and inactivated vaccines groups showed positive reactions against PPD-j that were significant compared to the PBS control quantified by an increase in skin thickness at the injection site at 72 h post injection (Figure 6). Interestingly, the reaction of pgsN-SC to PPD-b was minimal (1 out of 5 animals reacted to PPD-b) and not statistically different from the PBS control group while the inactivated group (4 out of 5 animals) reacted significantly to PPD-b (Figures 6A, B). The pgsN-IN group showed minimal skin test reaction that was not statistically different from PBS control group at both 3 and 6 MPV against both Johnin and PPD-b, suggesting that IN inoculation of pgsN does not elicit systemic cell mediated immunity, at least at the examined times.

To further dissect cell-mediated immunity following vaccination, we estimated the release of IFN-γ on monthly basis following immunization. As expected, both pgsN-SC and inactivated-SC groups induced significant levels of IFN-γ compared to the PBS group, especially at 2 MPV and forward (Figure 6C). On the other hand, the pgsN-IN group did not show any significant release of IFN-γ compared to the PBS control group. Finally, commercial ELISA indicated that only the inactivated-SC vaccine group elicited levels of antibodies above the basal levels from the 1 MPV timepoint and onward (Figure 6D). Taken together, SICST, IFN-γ release and ELISA indicated the biased induction of cellular immunity in pgsN-SC group.

## Discussion

Despite the previous availability of inactivated vaccine (Mycopar^®^) for cattle, the control of JD through vaccination has not been successful to date (31, 32). However, the inactivated vaccine was able to delay the clinical signs and decrease shedding of *M. paratuberculosis* in the environment (33). In Australia, >80% of vaccinated sheep flocks still shed *M. paratuberculosis* (34), risking the introduction of the disease to newly born animals. In a previous study by our group, the vaccine (pgsN) was shown to prevent shedding in challenged animals and elicited cell mediated immune responses (8). In the current study, we examined the nature of the immune responses elicited by pgsN in comparison to an inactivated vaccine, represented by the commercial Mycopar^®^ vaccine (discontinued by the manufacturer in 2020). A significant goal for this study was to examine visible granulomas formed by each vaccine type as a “mirror” to cellular recruitment elicited by live and inactivated vaccines in goats, a relevant model for studying Johne's disease in ruminants (20). We focused on the first 6 months of vaccination before interference from other agents that animals might face at later times. Fortunately, all parameters used to examine vaccine safety delivered by either subcutaneous or intranasal routes indicated that the live-attenuated vaccine was well-tolerated in animals. However, animals vaccinated with the inactivated vaccine formed well-organized, visibly large and caseating granulomas at the site of injection that persisted until 6 MPV, unlike pgsN-SC vaccinated animals. In addition, by 6 MPV, all animals in the inactivated vaccine group had granulomas at both prescapular (local) and mesenteric (distant) lymph nodes. Granulomas at injection sites and within prescapular lymph nodes of 2/5 animals displayed persistence of AFB. Granulomas at the site of vaccine injection in cattle usually trigger partial condemnation at slaughterhouses with associated reduced value for carcasses (15, 35). Thus, the subsiding of site-specific granulomas, with only 1 animal bearing a small palpable induration in the pgsN-SC group at 6 MPV, combined with absence of histologically evident intralesional AFB and lack of negative overall health effects, would result in less condemnation at slaughter and confirms the safety profile of the live vaccine.

Previously, an earlier format of the pgsN vaccine elicited robust T-cell immunity and prevented shedding in vaccinated goats and calves (8, 10). Granulomas formed at 3 WPV and 6 MPV, both are considered early times for the pathogenesis of Johne's disease (27, 36). Interestingly, granulomas associated with pgsN vaccination showed an initial, apparent delay in lymphocyte recruitment, in contrast, the 6 MPV timepoint had large nodular aggregates of predominantly B lymphocytes that were found to peripheralize the granuloma in the singular affected animal. Granulomas in the inactivated vaccine group were bordered by relatively equal proportions of CD3 + T and CD20 + B lymphocytes and similar numbers were maintained over time (at 3WPV and 6 MPV). Remarkably, CD3 is a known marker for T cells that are activated in response to *M. paratuberculosis* antigens (37) which needs further analysis that is beyond the scope of this study. This profile is contradictory to the profile generated in goats when another inactivated vaccine, Silirum, was evaluated showing CD20 + cells represented the lowest percentage of tissue immune cells (38). Unfortunately, the study goals did not include the analysis of circulating immune cells and rather focused on examining the cellular recruitment to granulomas, unlike the Silirum study. Needless to say, it is hard to link the presence of T-cells in formed granulomas to vaccine protective efficacy since both inactivated and live vaccines were shown a variable level of protection in goats and calves (8, 10). Altogether, these findings suggest that the novel pgsN vaccine induces an initial polarization of immune response toward cell mediated immunity that increases over time, with little involvement of humoral immunity concomitant with a lack of histologically identifiable AFB by 6 MPV. Although similar numbers of T and B lymphocytes were present in equal proportions (irrespective of time points), at the periphery of granulomas in animals receiving the inactivated vaccine, the persistence of these granulomas and intralesional bacteria suggests the difference in immune response elicited by the two vaccines is not related to their ability to recruit lymphocytes.

The field usage of inactivated vaccines is limited due to their interference with the diagnosis of bovine tuberculosis under field conditions (39, 40). In some experimental infections of calves with *M. paratuberculosis*, no cross reactivities with bovine tuberculosis antigens were found (40, 41). Unlike these reports, there was a significant cross reactivity to *M. bovis* antigens in the inactivated vaccine group while only minimal reaction (1 out of 5) among animals in the live-attenuated vaccine group, suggesting it will be more suitable for field application in areas where testing for bovine tuberculosis is practiced. Moreover, the intranasal administration of the live vaccine yielded no skin reaction, nor any systemic immune responses, suggesting inability of this route to provide protective immunity against Johne's disease. Earlier, oral delivery (another route for mucosal immunization) of live-attenuated vaccine resulted in better immunity (20) than the IN delivery used in this report, suggesting the importance of involvement of gut-associated lymphatic tissues in generating immune responses against JD. In summary, we have generated a marker-less live vaccine (pgsN) that was safe and elicited robust immune responses when administrated *via* the subcutaneous route but not intranasal route. Administration of this live vaccine was not associated with any negative health effects, unlike vaccination with the inactivated vaccine. More analysis of the cellular responses to this live vaccine in cattle is warranted to profile both resident and immune circulating cells.

## Data availability statement

The datasets presented in this study can be found in online repositories. The name of the repository and accession number can be found below: NCBI; PRJNA884360.

## Ethics statement

The animal study was reviewed and approved by University of Wisconsin-Madison Institutional Animal Care and Use Committee.

## Author contributions

Conceptualization, funding acquisition, resources, visualization, and supervision: AT. Methodology: MH, CH, C-wW, and YP. Validation, formal analysis, and writing—review and editing: MH, SA, and AT. Investigation: MH, CH, KN, and AT. Data curation and project administration: MH and AT. Writing—original draft preparation: MH. All authors contributed to the article and approved the submitted version.

## Supplementary material

Supplementary Figure 1 Acid-fast staining showing extracellular AFB present within the caseating center of a prescapular lymph node in the inactivated vaccine group at 6 MPV (bar = 200 μm), similar to the inset in Figure 5B.
