# Supplementary figures and images for: Characterization of early immune responses elicited by live and inactivated vaccines against Johne's disease in goats
Source: Front Vet Sci. 2023 Jan 9;9:1046704. doi: 10.3389/fvets.2022.1046704 (PMC9868903; doi:10.3389/fvets.2022.1046704)

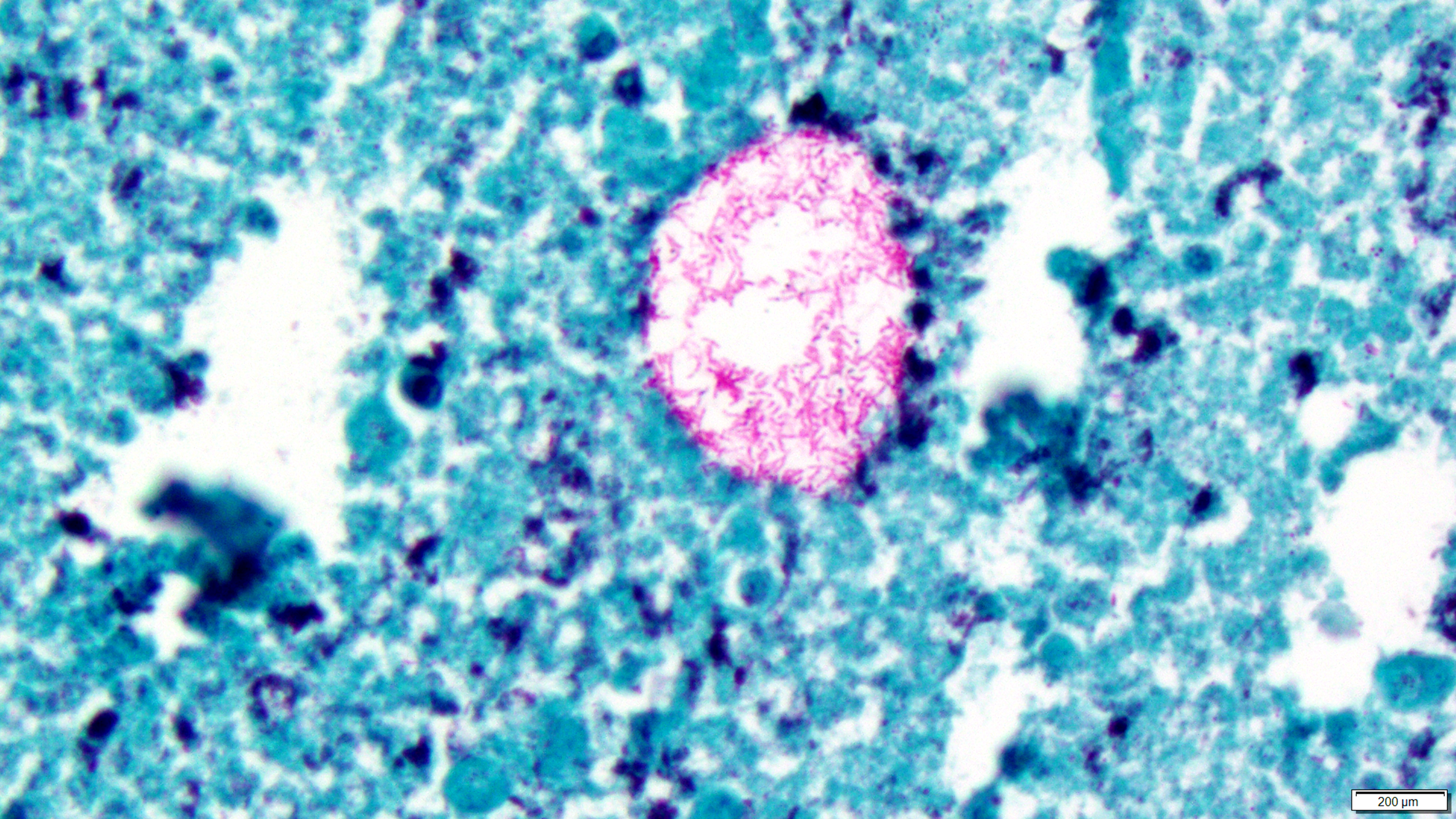

Supplement: Supplementary Figure 1 — Acid-fast staining showing extracellular AFB present within the caseating center of a prescapular lymph node in the inactivated vaccine group at 6 MPV (bar = 200 μm), similar to the inset in Figure 5B. [file Image_1.TIF]
